# Determinants of intra-specific variation in basal metabolic rate

**DOI:** 10.1007/s00360-012-0698-z

**Published:** 2012-07-31

**Authors:** Marek Konarzewski, Aneta Książek

**Affiliations:** Institute of Biology, University of Białystok, Świerkowa 20B, 15-950 Białystok, Poland

**Keywords:** BMR, Intra-specific variation, Quantitative genetics, Genomics, Artificial selection, mTOR

## Abstract

Basal metabolic rate (BMR) provides a widely accepted benchmark of metabolic expenditure for endotherms under laboratory and natural conditions. While most studies examining BMR have concentrated on inter-specific variation, relatively less attention has been paid to the determinants of within-species variation. Even fewer studies have analysed the determinants of within-species BMR variation corrected for the strong influence of body mass by appropriate means (e.g. ANCOVA). Here, we review recent advancements in studies on the quantitative genetics of BMR and organ mass variation, along with their molecular genetics. Next, we decompose BMR variation at the organ, tissue and molecular level. We conclude that within-species variation in BMR and its components have a clear genetic signature, and are functionally linked to key metabolic process at all levels of biological organization. We highlight the need to integrate molecular genetics with conventional metabolic field studies to reveal the adaptive significance of metabolic variation. Since comparing gene expressions inter-specifically is problematic, within-species studies are more likely to inform us about the genetic underpinnings of BMR. We also urge for better integration of animal and medical research on BMR; the latter is quickly advancing thanks to the application of imaging technologies and ‘omics’ studies. We also suggest that much insight on the biochemical and molecular underpinnings of BMR variation can be gained from integrating studies on the mammalian target of rapamycin (mTOR), which appears to be the major regulatory pathway influencing the key molecular components of BMR.

## Introduction

Basal metabolic rate (BMR) quantifies the minimum rate of energy expenditure necessary to maintain energy balance of resting, post-absorptive endotherms at thermoneutral conditions (Schmidt-Nielsen 1997). Although conceived with biomedical research in mind, BMR has quickly become the most widely used benchmark metric of metabolic rate (White and Kearney 2012). It has also become clear that the composition and variation in BMR convey extremely important biological information (for an extensive review see McNab 2002). Consequently, disentangling the factors and mechanisms that underlie differences in BMR at inter- and intra-specific level has become the key component of major questions at the interface between evolution, ecology, and physiology of endotherms, including the evolution of endothermy itself (Hayes 2010; Nespolo et al. 2011).

Historically, studies on variation in BMR were guided by the Krogh principle (1916), taking advantage of the several-fold inter-specific variation in BMR (McNab 2002). Although inter-specific studies have greatly contributed to our understanding of the general patterns of BMR variation, they are limited by two important factors. First, studies at inter-specific level must take into account the potentially confounding effect of phylogeny on the inference (Garland et al. 1999). Second, inter-specific analyses are based on the assumption that a species-specific average BMR value used in those analyses adequately characterizes BMR at the intra-specific level. This assumption is legitimate, when inter-specific variation widely exceeds intra-specific variation (Ives et al. 2007). It must be borne in mind, however, that it is intra-specific variation that is a substrate of natural selection, and therefore, inter-specific studies on BMR can only partially inform the inference on adaptation and cannot unambiguously identify factors influencing its variation. For this reason, patterns derived from inter-specific analyses cannot be directly extrapolated to the intra-specific level. On the other hand, however, intra-specific studies on metabolic traits also have their limitations. The most important one is the narrow range of within-species variation, which hampers the power of statistical analysis (Konarzewski et al. 2005). This limitation is exactly why researchers often resort to inter-specific comparisons, even though they do not always provide sufficient methodological justification for the inferences they make (Garland and Adolph 1994).

Despite the limited statistical power, studies on intra-specific variation in BMR become increasingly attractive thanks to the wealth of information on the molecular underpinnings of energy expenditure, which by definition are more applicable at the intra- than the inter-specific level of inference (Stapley et al. 2010). Also, the state-of-the-art equipment used to quantify metabolic rates now offers the possibility to reduce the measurement error of BMR down to 15 % (Konarzewski et al. 2005, for an extensive review of measurement techniques see Lighton 2008), which most likely underlies the increasing number of studies reporting statistically significant within-species repeatability of BMR (for review see Nespolo and Franco 2007). Here, we decompose variation in BMR and review the literature pertaining to different levels of biological organization that contribute to variation in BMR. In doing so we have exclusively focused on body mass-corrected BMR, because whole-body BMR and its relation to body mass have been recently reviewed (White and Kearney 2012). BMRs discussed herein have been analysed by one of general linear models (most often by ANCOVA with whole-body mass as a covariate), which effectively accounts for the effect of body mass and allows for a direct comparison of individuals of different masses. Following intense debate (Packard and Boardman 1987; Tracy and Sugar 1989; Jasienski and Bazzaz 1999) such statistical means of correction of body mass have become widely accepted by integrative physiologists. It is important to note in this context, that the controversy regarding how best to control for the impact of body mass on physiological traits still remains unsettled in biomedical literature (Kaiyala and Schwartz 2011).

Also, numerous studies (e.g. Szafrańska et al. 2007) do not meet the criteria that animals are quantified in a post-absorptive state, which is part of the definition of BMR. However, as meeting this condition most likely does not appreciably affect the determinants of BMR discussed in this review (e.g. the contribution of organ sizes to BMR), we equate resting metabolic rate (RMR) to BMR.

We start by decomposing BMR variation at the organ, tissue and molecular level. We mainly base our review on mammalian studies, as the majority of the relevant information presented herein comes from research on laboratory rodents and humans. However, wherever possible, we also highlight how the results of laboratory studies can be applied to free-ranging animals, though we do not extensively discuss BMR in an ecological context, as it has been recently reviewed elsewhere (Burton et al. 2011). Finally, we review recent advancements in the quantitative genetics of BMR and organ mass, as well as the molecular genetics of BMR.

## Composition of BMR at organ level

At its most fundamental level, whole-body BMR is the sum of the products of organ masses and their mass-specific metabolic rates (Schmidt-Nielsen 1984; Wang et al. 2001). Rodents, in particular laboratory mice and rats, are undoubtedly the best studied animal models with regard to both of these components, and consequently, their contribution to BMR. Visceral organs (heart, kidney, liver, and small intestine) and the brain that are primarily responsible for energy flux comprise ~5–8 % of body mass of laboratory rats and mice, as well as humans (Müller et al. 2002). Konarzewski and Diamond (1995) tested the intra-specific correlation between BMR and the masses of four visceral organs (heart, kidney, liver, and small intestine) among six inbred strains of laboratory mice. They found that strains with exceptionally high (or low) BMR tended to have disproportionately large (or small) organs. Their combined mass accounted for 52 % of the variation in BMR. Likewise, Sacher and Duffy (1979) found a positive correlation between BMR and brain mass in laboratory mice. However, several studies carried out on an outbred MF1 strain of laboratory mice have not found significant correlations between BMR and internal organ masses (Johnson et al. 2001a, b; Król et al. 2003). Such correlations were also absent in studies carried out on several wild rodents (e.g. white-footed mouse, Koteja 1996; leaf-eared mouse, Nespolo et al. 2003 and Bacigalupe et al. 2004; Mongolian gerbils, Chappell et al. 2007). On the other hand, biomedical research unequivocally indicates that internal organs significantly contribute to human BMR. This research builds upon the quickly advancing development of imaging techniques, particularly computer tomography and magnetic resonance imaging (MRI), allowing for the quantification of the organ size in vivo and their contribution to variation in human BMR (e.g. Later et al. 2008; Javed et al. 2010; Müller et al. 2002, 2011). For example, Elia (1992) estimated the mass-specific metabolic rates (in kcal/kg per day) of major human organs of young adults to be: 200 for liver, 240 for brain, 440 for heart and kidneys, 13 for skeletal muscle, 4.5 for adipose tissue and 12 for residual mass. These estimates were recently validated with the use of imaging technologies, which also allowed for the fine-tuning of Elia’s estimates with respect to the effect of gender differences (Wang et al. 2011a) and obesity (Wang et al. 2011b). Likewise, a recent imaging-based comprehensive analysis of the scaling of human BMR and organ masses revealed that muscle, brain and liver explained up to 43 % of the inter-individual variance in human BMR (Müller et al. 2011).

Theoretically the most robust test of the existence of a positive association between BMR and metabolically expensive organ masses should be provided by artificial selection experiments aimed at either BMR or the masses of those organs. Assuming that there exists sufficient additive genetic variation, such experiments allow for the change of frequencies of alleles directly related to energy expenditures (either at the level of the mass-specific metabolic rates or whole organs). The major advantage of an experimental manipulation of BMR is its explanatory ability to distinguish non-causative correlations between BMR and anatomy from biologically meaningful, inescapable links underlined by the genetic architecture of postulated associations (for an extensive review of artificial experiments on rodents see Swallow et al. 2009). If the postulated, inexorable link between BMR and organ masses exists, then such selection should result in concerted unidirectional changes in both directly selected and secondary (correlated) traits (for theoretical justification see Hayes 2010).

Several artificial selection experiments on rodents have achieved substantial changes in BMR (Książek et al. 2004) and other metabolic traits including maximum metabolic rate (Middleton et al. 2008; Wone et al. 2009), body mass-corrected food intake (Bunger et al. 1998), heat loss (Nielsen et al. 1997a, b) or food digestibility (Sadowska et al. 2008). We summarized their major findings in Table 1. Among these experiments only one directly selected on BMR, resulting in a conspicuous 40 % difference in BMR (quantified as residuals from a regression of BMR on body mass) between low and high selected line types, derived from Swiss Webster outbred strain of laboratory house mice (Książek et al. 2004, 2009; Konarzewski et al. 2005; Brzęk et al. 2007; Gębczyński and Konarzewski 2009; for review see Swallow et al. 2009). This between-line difference in BMR was also reflected in considerable differences in internal organ masses: mice from the high-BMR line had larger hearts, livers, kidneys, and small intestines (Książek et al. 2004; Książek and Konarzewski 2012). Those differences between the organ sizes in high and low line types ranged from 14 % for hearts, 17 % for livers, 18 % for kidneys and 13 % for small intestines in generation F19, and increased to 16, 18, 31 and 34 %, respectively, in generation F31. The resulting differences were significantly larger than divergences that could result from random genetic drift alone, and thus support the existence of a genuine genetic correlation between organ masses and BMR.

**Table 1:** Summary of the responses to artificial selection on metabolic and related traits in rodents

Selection criterion/method/species	BMR response	Correlated traits	Trait response	Reference
Mass-corrected BMR/indirect calorimetry/laboratory mice (*Mus musculus*)	Increase	Food consumption	Increase	Książek et al. (2009)
		Voluntary activity	Increase	Gębczyński and Konarzewski (2009)
		*V*O_2max_ (treadmill)	No change	
		*V*O_2max_ (swim elicited)	Decrease	Książek et al. (2004)
				Brzęk et al. (2007)
		Core body temperature	No change	Gębczyński (2008)
				Brzęk et al. (2012)
		Mass of heart, liver, kidney, small intestine	Increase	Książek et al. (2004)
				Gębczyński and Konarzewski (2011)
		Fat mass	Decrease	Książek et al. (2004)
		BAT mass	Decrease	
		Erythrocyte size	Decrease	Maciak et al. (2011)
		Immune response (SRBC)	Decrease	Książek et al. (2003)
		Immune response (KLH)	Increase	Książek and Konarzewski (2012)
		Mass of spleen and lymph nodes	Increase	
		Thymus mass	Decrease	
		Oxidative enzyme capacity	Increase	Książek et al. (2009)
		Unsaturation index of cell membranes	Decrease	Brzęk et al. (2007)
Mass-corrected food intake/laboratory mice (*Mus musculus*)	Increase	Digestive efficiency	Increase	Hastings et al. (1997)
		Fat mass	Decrease	Bunger et al. (1998)
		Core body temperature	No change	Hambly et al. (2005)
		Liver mass (dry)	Increase	
		Small intestine length (fresh)	Increase	
		Small intestine mass (dry)	No change	Selman et al. (2001a, b)
		Large intestine mass (dry)	Decrease	
		Pancreas mass (dry)	No change	
		Stomach mass (dry)	Increase	
		Kidneys mass (dry)	No change	
		Heart mass (dry)	Increase	
		Lung mass (dry)	No change	
		Brain mass (dry)	Increase	
		Thyroid mass (dry)	Decrease	
		Spleen mass (dry)	No change	
Heat loss/(body mass)^0.75^/direct calorimetry/laboratory mice (*Mus musculus*)	Not measured	Food consumption	Increase	Nielsen et al. (1997b)
		Voluntary locomotor activity	Increase	Nielsen et al. (1997a)
		Mass of liver, heart, spleen	Increase	Moody et al. (1999)
		Core body temperature	Increase	Mousel et al. (2001)
		T4 level	Decrease	Kgwatalala and Nielsen (2004)
		T3 level	No change	
		Corticosterone level	Increase	
		Expression of UCP-1	Decrease	McDaneld et al. (2002)
Mass-corrected *V*O_2max_/swimming/laboratory mice (*Mus musculus*)	No change	Heart mass	Increase	Gębczyński and Konarzewski (2009)
		Mass of liver, kidney, small intestine	No change	
		Mass of gastrocnemius	Increase	
Aerobic endurance capacity/treadmill running/rats (*Rattus norvegicus*)	Not measured	Body mass	Decrease	Koch and Britton (2001)
		Fat mass	Decrease	Kirkton et al. (2009)
		*V*O_2max_	Increase	Henderson et al. (2002)
		Mass of heart, lung, liver, kidney, stomach	Increase	Swallow et al. (2010)
		Cardiac output	Increase	
		Pulmonary function	Increase	Howlett et al. (2003)
		Oxidative enzyme capacity	Increase	
		Left ventricular cells systolic and diastolic function	Increase	
		Small intestine length	Decrease	Wislöff et al. (2005)
		Capillary density	Increase	Henderson et al. (2002)
		Mitochondrial biogenesis	Increase	Gonzales et al. (2006)
		Oxidative enzyme capacity	Increase	Wislöff et al. (2005)
Voluntary locomotor activity/daily wheel running activity/laboratory mice (*Mus musculus*)	No change	*V*O_2max_	Increase	Swallow et al. (1998)
				Rezende et al. (2006a, b, c)
				Kane et al. (2008)
		Body mass	Decrease	Middleton et al. (2008)
		Fat mass	Decrease	Girard et al. (2007)
				Vaanholt et al. (2007)
		Muscle mass	Decrease	Guderley et al. (2008)
				Middleton et al. (2008)
		Heart (ventricle), spleen, liver, adrenal glands	No change	Swallow et al. (2005)
		Corticosterone levels	Increase	Malisch et al. (2009)
Three-way selection/bank vole *Myodes* (*Clethrionomys*) *glareolus*				
*V*O_2max_ (swim elicited)	Increase	Food consumption	Increase	Sadowska et al. (2008)
		Core body temperature	Increase	P. Koteja, unpublished
Ability to grow on a low-quality herbivorous diet	No change			
Intensity of predatory behaviour	No change			
Mass-corrected *V*O_2max_/treadmill running/laboratory mice (*Mus musculus*)	Increase	The liver amino acids and tricarboxylic acid cycle (TCA cycle) metabolites	Decrease	Wone et al. (2011)
		Gastrocnemius, amino acids and TCA cycle metabolites	Increase	

The results of direct selection on BMR are complementary to those of other artificial selection experiments, which have targeted traits closely correlated with BMR. Selman et al. (2001a, b) showed that artificial selection of laboratory mice for a high rate of food consumption resulted in both larger sizes of the internal organs and BMR, compared with mice from lines selected for a low rate of food consumption (Table 1). Nielsen et al. (1997a, b) selected laboratory mice for high and low heat loss, measured in adult males during a 15-h assay using a direct calorimetry system. This selection too resulted in larger metabolically active organs (liver and heart) in line types selected for high heat loss, as compared to the control line types (Kgwatalala and Nielsen 2004). Overall, the results of artificial selection experiments provide mounting evidence for the existence of a strong genetic correlation between BMR (or related metabolic rates) and the masses of energetically expensive internal organs. Recall, however, that those correlations account for ca. 50 % BMR variation (Konarzewski and Diamond 1995). It is also important to note that measurements of BMR are technically complicated and inherently burdened with measurement error of 15–20 % (Konarzewski et al. 2005; Lighton 2008). This still leaves ca. 30 % of BMR variation not accounted for. It is very likely that the remaining variation can be attributed to fat and/or muscle mass. Indeed, the inverse correlation between fat mass and BMR has been reported in many artificial selection studies (e.g. Bunger et al. 1998; Książek et al. 2004; see Table 1). On the other hand, specific studies on the contribution of muscle mass to intra-specific variation in BMR in small mammals have not been carried out. Interestingly, recent metabolomic analysis of metabolite profiles of mice selected for mass-corrected maximum metabolic rate suggest that BMR may increase due to elevated amino acid and energy metabolism in the musculature (Wone et al. 2011). Further such study would be very desirable, as at the inter-specific level, muscle mass, but not internal organ mass seems to explain most of the variation in BMR (Raichlen et al. 2010).

## Composition of BMR at the tissue and molecular levels

At rest, the mass-corrected metabolic rate of endotherms is 5–10 times higher than the mass-corrected metabolic rate of ectotherms (Bennett and Ruben 1979; Hulbert and Else 1981). At the cellular level, the major process that accounts for this difference is mitochondrial uncoupling and proton leak across the inner mitochondrial membrane, in mammals largely mediated by mitochondrial carrier proteins (Porter et al. 1996; Dulloo and Samec 2001). The most prominent of them is uncoupling protein-1 (UCP-1), which facilitates non-shivering thermogenesis in mammalian brown adipose tissue (BAT; Cannon and Nedergaard 2010). According to the ‘membrane pacemaker’ theory of metabolism, the key factor behind the ‘leakiness’ of the cell membranes is the chemical composition of their fatty acids, particularly the relative abundance of long-chain polyunsaturated fatty acids (PUFAs; Hulbert 2003; Hulbert and Else 1999, 2000, 2005). The chemical composition of fatty acids affects the physical properties of cell membranes, which in turn modulates the activity of many metabolically important enzymes and determines metabolic costs of maintenance of ionic gradients across cell membranes (Else and Wu 1999; Wu et al. 2001). Indeed, differences in fatty acyl composition of the mitochondrial membranes and in proton leak between ecto- and endotherms are well documented (e.g. Mitchell et al. 2007). However, their contribution to inter-specific variation in BMR in mammals is less clear: proton leak does not explain differences in BMR between marsupials and eutherians (Polymeropoulos et al. 2012), and there is no notable correlation between mammalian BMR and muscle phospholipid fatty acid composition (Valencak and Ruf 2007).

Direct evidence for the quantitative contribution of proton leak to within-species variation in BMR comes from two studies. Rolfe and Brown (1997) reported that as much as 20 % of BMR can be attributed to an incomplete coupling of substrate oxidation. It is therefore likely that a significant proportion of BMR variation is mediated through UCPs. This was demonstrated by Speakman et al. (2004) who found that individual MF1 mice having high BMR also have skeletal mitochondria characterized by high proton conductance. This increased conductance was caused by higher levels of endogenous activators of proton leak through the adenine nucleotide translocase and uncoupling protein-3 (UCP-3). On the other hand, however, McDaneld et al. (2002) found that a response to selection for increased energy expenditure in mice selected for heat loss by Nielsen et al. (1997a, b) was not mediated by increased expression or function of UCP-1 (for detailed characterization of this selection experiment see Table 1). Contrary to expectations, the mice in the low heat-loss line expressed significantly more UCP-1 mRNA than did high heat-loss mice. McDaneld et al. (2002) also found that uncoupling protein-2 (UCP-2) mRNA content was similar in mice characterized by high and low heat loss. Thus, conspicuous differences in energy expenditure can be independent of UCP-1 and UCP-2-mediated thermogenesis.

There is also no direct evidence for a correlation between cell membrane fatty acid composition and BMR within species as predicted by the ‘membrane pacemaker’ theory. Haggerty et al. (2008) found no correlation between BMR and lipid desaturation in the liver of MF1 mice. They also did not find a correlation between BMR and either the proportion of oleic acid (18:1) or highly polyunsaturated 22:6 docosahexanoic (DHA) fatty acid content in liver membranes. The lack of a correlation between BMR and the proportion of DHA is particularly telling because DHA has been identified as key component of the ‘membrane pacemaker’ theory (Hulbert and Else 1999, 2000, 2005). Brzęk et al. (2007) analysed cell membrane fatty acyl composition in the liver and kidneys of mice divergently selected for BMR. Contrary to the predictions derived from the ‘membrane pacemaker’ theory the unsaturation index (the number of double bonds per 100 acyl chains) of the fatty acids in the kidney cell membranes did not differ between selected lines, despite 30 % difference in BMR. Furthermore, the unsaturation index was higher in livers of mice from the low-BMR line, mainly because of a significantly higher content of DHA. Thus, divergent selection for BMR affected fatty acyl composition of phospholipids in the liver in the opposite direction to that predicted by the ‘membrane pacemaker’ theory. It is important to note, however, that the lack of support for this theory does not necessarily question the contribution of the proton leak to BMR.

Apart from proton leak, the activity of the Na–K ATPase for the maintenance of cell membrane ionic gradients has been implicated as another component of BMR, accounting for ca. 20 % of its variation (Rolfe and Brown 1997; Wu et al. 2001). Nevertheless, it is unclear how much of the costs of maintaining ionic gradients contribute to intra-specific variation in BMR. The cell metabolism hypothesis (Kozłowski et al. 2003) suggests an intriguing, yet untested, functional link between those costs and variation in BMR, mediated through variation in the size of individual cells constituting tissues and organs. It follows from the simple geometric relationship between surface area and volume that individuals of similar body mass, but built of larger cells should have relatively smaller cell summed surfaces than those, built of smaller, but more numerous cells. Thus, all else being equal, a simple way to decrease/increase BMR is to decrease/increase the cell size of metabolically expensive tissues. To our knowledge, there are no published studies that allow for a direct test of this hypothesis in homeotherms. Maciak et al. (2011) tested it indirectly by comparing the mass-corrected standard metabolic rate (SMR) and erythrocyte size (used as a proxy for cell size, for justification see Kozłowski et al. 2010) between diploid and triploid individuals of a small fish belonging to the *Cobitis* *taenia* hybrid complex. Triploids within this complex have 2.3 larger erythrocytes due to the effect of ploidy. Maciak et al. (2011) demonstrated an inverse correlation between cell size and SMR. Recently, Maciak also found a similar inverse relationship between BMR and erythrocyte size in lines of mice divergently selected for BMR, with H-BMR individuals having 10 % smaller erythrocytes than those of L-BMR line (Maciak, unpublished PhD thesis). It is important to note in this context that the ploidy level, and therefore cell size, of metabolically expensive organs, such as liver, can vary within mammalian species, including humans (Duncan et al. 2010). These observations provide empirical support for cell size as an important determinant of variation in BMR at the intra-specific level, though its generality remains to be established (Kozłowski et al. 2010).

**Fig. 1:**
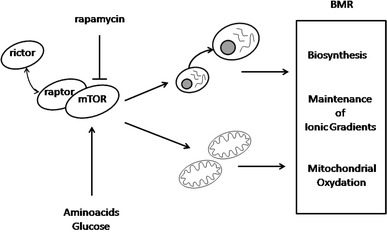
Schematic representation of regulation of BMR variation by the mTOR pathway. The mTOR–raptor complex responds to nutrient availability by up- or down-regulating mitochondrial oxidation. It also controls cell growth, which in turn generates metabolic costs of biosynthesis directly, and indirectly affects the metabolic costs of maintenance of the membrane ionic gradients being the function of the cell size

**Fig. 2:**
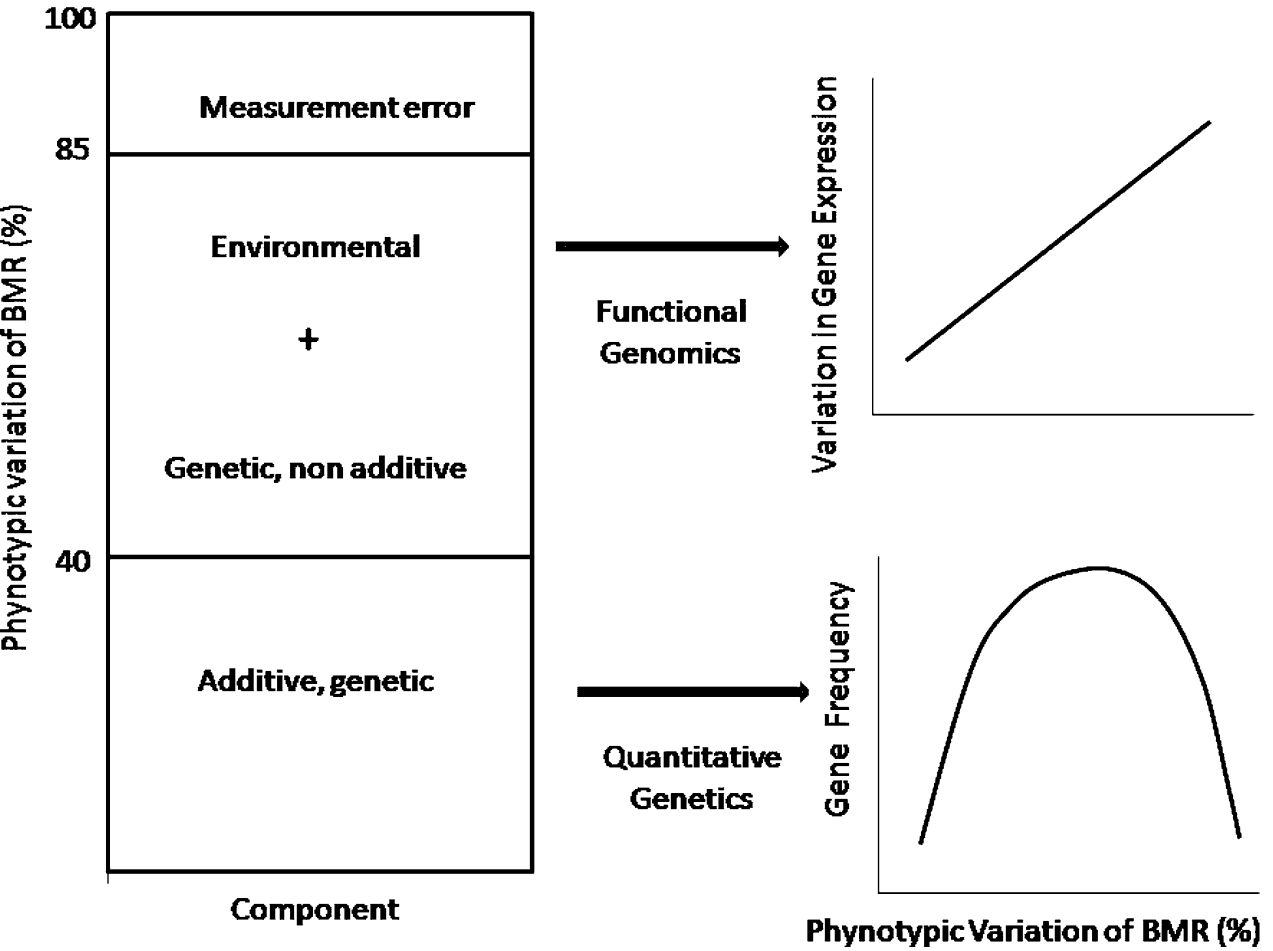
Schematic representation of phenotypic variation in BMR. Quantitative genetics studies indicate that ca. 40 % of phenotypic variance can be attributed to additive genetic effects (Table 1 in White and Kearney 2012). Thus, it is likely that in most populations the frequency of alleles underlying BMR is somewhere between two extremes: (1) the loss of genetic variation due to genetic drift or purifying selection, (2) the fixation of alleles due to long-term directional selection. Assuming a 15 % measurement error of BMR (Konarzewski et al. 2005), ca. 45 % of the total BMR variation can be due to environmental effects and non-additive gene expression. This points to the need to examine BMR variation using functional genomics tools

Besides proton leak and the maintenance of ionic gradients, the third most important component of cellular metabolism is the cost of biosynthesis, comprising ca. 20 % of BMR (Rolfe et al. 1999). Interestingly, all three components are regulated by a single signalling pathway—the mammalian target of rapamycin (mTOR), which is therefore likely to be the most important molecular mechanism underlying the within-species variation in BMR (Fig. 1; for review see Laplante and Sabatini 2009; Ramanathan and Schreiber 2009; Schieke et al. 2006). The mTOR is a serine/threonine protein kinase coded by a highly conserved TOR gene found within every eucaryote genome (Wullschleger et al. 2006). mTOR forms two distinct complexes: (1) mTORC1, which is inhibited by antibiotic rapamycin and contains the protein component called raptor (Fig. 1), and (2) mTORC2 insensitive to rapamycin, and in which mTOR is bound to another protein partner called rictor (Wullschleger et al. 2006). The pivotal role of mTOR in cell size and growth regulation is well documented (Guertin et al. 2006). The main environmental cue affecting mTOR activity is the availability of nutrients, mostly amino acids and glucose. In the absence of amino acids the mTOR signalling is inhibited and protein synthesis is thereby down-regulated, which arrests cell growth. Cell metabolism is mainly regulated by mTORC1 through AMP-activated protein kinase (AMPK), which is in turn activated in response to a high AMP/ATP ratio within cells. Schieke et al. (2006), used Jurkat T cell leukaemia clone E6-1 cells as a model and found a positive correlation between resting mitochondrial respiration of individual cells and the activity of their mTOR–raptor complexes. They also demonstrated that inhibition of mTORC1 by rapamycin administration leads to the reduction of mitochondrial membrane potential, oxygen consumption, and consequently ATP production. According to Schieke et al. (2006), mTOR activity is also likely to determine the balance between generation of ATP through mitochondrial and non-mitochondrial cascades.

## Quantitative genetics of BMR

High levels of BMR underlying endothermy have most likely evolved as a correlated response to selection for high rates of aerobic metabolism (Bennett and Ruben 1979) or an increased parental investment capacity (Koteja 2000). Theoretical analyses of the genetics of the evolution of endothermy provide opposing predictions with regard to determination of BMR and its covariation with other physiological traits. According to a ‘strong’ version of the aerobic capacity model a positive genetic correlation between BMR and other traits (chiefly maximum metabolic rate, MMR) is an inexorable feature of the design of homeotherms, and therefore persisted not only at the early stages of BMR evolution, but is also present in extant birds and mammals (Hayes 2010; Nespolo et al. 2011). On the other hand, however, the ‘weak’ form the aerobic capacity model predicts that directional selection was likely to result in the fixation of genes conferring a phenotypic advantage and, consequently, has resulted in a considerably reduced genetic variation of BMR and its covariation between traits (Nespolo et al. 2011). According to this evolutionary scenario, genetic variation and covariation of BMR that was present in proto-endotherms may no longer be detectable in some or all extant lineages of homeotherms by means of classic methods of quantitative genetics.

Despite fundamental significance of the question of the extent of genetic determination of BMR, to date the great majority of studies on variation in BMR have focused on its phenotypic variation and discussed its adaptive significance based solely on non-genetic data (e.g. McNab 2002). It is important to note, however, that phenotypic variation per se does not allow for meaningful evolutionary inference (Roff 2002). Likewise, phenotypic correlations between studied traits do not necessarily reflect their potential to respond to selection in a concerted fashion, as the strength (and even sign!) of phenotypic and genetic correlations may differ (Roff 2002). Only recently integrative physiologists have become aware of the need to study heritable variation in physiological traits (Nespolo et al. 2003; Sadowska et al. 2005; for review see Swallow et al. 2009). For this reason, the number of studies on BMR heritability is limited and primarily restricted to classical laboratory model rodents (Dohm et al. 2001; Konarzewski et al. 2005) or wild species bred under laboratory conditions (Nespolo et al. 2003, 2005; Sadowska et al. 2005; Rønning et al. 2007).

A key quantitative genetic parameter, informing the potential of a given trait to respond to selection and therefore, to evolve, is the narrow-sense heritability (*h* ^2^), which is the ratio of additive genetic variance to total phenotypic variance (Falconer and Mackay 1996). Early laboratory studies indicated very low or even insignificant narrow-sense heritability of BMR in laboratory mice (Lacy and Lynch 1979; Lynch and Sulzbach 1984; Dohm et al. 2001) and a wild rodent, the leaf-eared mouse *Phyllotis darwini* (Nespolo et al. 2003; Bacigalupe et al. 2004). Furthermore, the heritability of some traits closely related to BMR, such as body temperature, is also effectively zero (Lacy and Lynch 1979; Lynch and Sulzbach 1984; Nespolo et al. 2003). More recent studies, however, have found a relatively high and significant narrow-sense heritability of BMR in laboratory mice (*h* ^*2*^ = 0.38, Konarzewski et al. 2005; *h* ^*2*^ = 0.19, Wone et al. 2009), bank voles *Myodes* (*Clethrionomys*) *glareolus* (*h* ^*2*^ = 0.4, Sadowska et al. 2005) zebra finches *Taeniopygia guttata* (*h* ^*2*^ = 0.25, Rønning et al. 2007) and least weasels (*Mustela nivalis*, Zub et al. 2012, for detailed review see White and Kearney 2012). Thus, it seems that at least in some animal populations there is substantial additive genetic variation in BMR at the level of ca. *h* ^*2*^ ~0.4 (Fig. 2). Also, the presence of a significant genetic component of human BMR was supported by studies on families participating in phase 2 of the Québec Family Study (Rice et al. 1996; Jacobson et al. 2006), which is likely related to polymorphisms in the leptin and leptin receptor genes (Loos et al. 2006).

Despite the unquestionable advantages of laboratory conditions, these studies yield estimates obtained in an artificial environment, and on animals that are more inbred than individuals in natural populations. Constant laboratory-controlled conditions are also likely to inflate heritability estimates, because the effect of environmental variation is lower than in natural populations (Riska et al. 1989). On the other hand, the propensity of BMR to be influenced by environmental factors such as temperature, food availability and photoperiod (McNab 2002) make field estimates of BMR *h* ^*2*^ very difficult. Studies examining the heritability of metabolic traits in wild populations are even more complicated by difficulties associated with constructing a pedigree, which is necessary for calculating *h* ^*2*^ (Lynch and Walsh 1998; Coltman 2005).

To date there are just two published studies estimating the *h* ^*2*^ of BMR in the wild. Nilsson et al. (2009) found a significant *h* ^*2*^ of BMR in a small, cavity-nesting passerine—the blue tit (*Cyanistes* *caeruleus*). This study used split cross-fostering of nestlings, which allowed for an effective separation of genetic and environmental effects on BMR for individuals in a known pedigree. Unfortunately, for most wild populations information on environmental factors affecting individuals and their relatedness is unavailable. Pedigree reconstruction, however, has now become possible thanks to the application of methods utilizing inference derived from the analysis of highly polymorphic molecular markers (for review see Garant and Kruuk 2005; Pemberton 2008). Thus, the reconstructed structure of relatedness can be combined with individual BMR measurements and analysed using a class of statistical analysis referred to as the ‘animal model’ (for review see Kruuk 2004; Shaw 1987; Thompson 2008). The animal model is based on restricted maximum likelihood (REML) computational techniques and consists of a mixture of both ‘fixed’ and ‘random’ effects, which allows for the effective partitioning of the phenotypic and genetic components of variance (Wilson et al. 2009).

We are aware of one successful attempt utilizing the approach outlined above. Zub et al. (2012) used a marker-based approach to reconstruct the pedigree and then used an ‘animal model’ to estimate the *h* ^*2*^ of body mass and BMR in the free-living population of weasels *Mustela nivalis*—a small carnivore characterized by a wide range of body mass and extremely high BMR. Zub et al. (2012) found that the *h* ^*2*^ of whole-body BMR and BMR was equal to 0.54 and 0.45, respectively, which are values comparable to laboratory *h* ^*2*^ estimates. The only environmental factor affecting the *h* ^*2*^ estimate of BMR was seasonal variation. The study of Zub et al. (2012) demonstrates clearly that marker-based approaches to pedigree reconstruction make it possible to analyse data for metabolic traits in wild populations. Such data would otherwise be impossible to obtain in the absence of pedigree information.. This has become exceptionally important in the context of microevolutionary responses to climate change and the paucity of data for disentangling the genetic (evolutionary) and phenotypic (plastic) components of physiological mechanisms underlying those responses (for review see Gienapp et al. 2008; Feder et al. 2010).

## Genomics of BMR

The results of quantitative genetic analyses strongly suggest that a significant part of the phenotypic variance in BMR can be attributed to an additive genetic component, mostly underlined by many genes of small effect, coding for structural polymorphism of proteins. However, another ca. 40 % of BMR variation can be attributed to environmental effects and non-additive genetic effects, presumably acting through the modulation of gene expression (Fig. 2; Nespolo et al. 2011; for a concise review of the concepts see Whitehead and Crawford 2006). The relative contribution of genes underlying structural polymorphism and gene expression to the overall genetic variation of BMR remains to be determined. However, there is a mounting body of evidence that many phenotypic differences within and between populations are due to differences in gene expression (e.g. Oleksiak et al. 2002). This might be the case for BMR, if underlying metabolic pathways were conserved in the course of its evolution and are mainly determined by the degree of expression of genes shared by ecto- and endotherms (Seebacher et al. 2006; Schwartz et al. 2008). Although this still remains to be tested, the advent of a new generation of DNA sequencing and gene expression technologies brings the promise of a rapid progress in understanding the genetic/genomic basis of complex physiological traits, such as BMR (e.g. Vera et al. 2008; Stapley et al. 2010; Wheat 2010).

To date no genomic studies have specifically examined BMR. Nevertheless, there is already a wealth of information available on the genomics of traits that likely contribute to BMR, such as the mapping of QTLs underlying aerobic capacity (Moody et al. 1999; Jacobson et al. 2006) and the examination of gene expression in metabolically expensive tissues (Klaus et al. 2005). The scope of this paper prevents us from a detailed appraisal of the genomics of metabolic rates, which certainly deserves a dedicated review. We have therefore decided to concentrate on the skeletal and heart muscles because many studies suggest that they significantly contribute to BMR (Konarzewski and Diamond 1995; Raichlen et al. 2010), and focus on recent insights gleaned from artificial selection experiments and transgenic manipulations.

One of the best studied models are mice (Garland 2003; Middleton et al. 2008) and rats (Koch and Britton 2001; Wislöff et al. 2005) divergently selected for endurance capacity (Table 1). Koch and Britton’s experiment rats were divergently selected for exercise capacity by treadmill running at 11 weeks of age. Given that the line selected towards high endurance running capacity was characterized by increased: (1) food consumption, (2) percent lean mass and (3) mass of metabolically active visceral organs (Swallow et al. 2010), it seems reasonable to assume that the selection also resulted in a between-line divergence in BMR. However, despite the 120 % between-line difference in the primary selected trait, Bye et al. (2008a) found only three differences in the expression of transcripts in the soleus muscle of rats of both lines, with unclear, immediate connection to BMR. Much more conspicuous genetic differences have been found among lines of laboratory mice selected for increased levels of voluntary wheel running (Middleton et al. 2008). Mice from two of the four selected replicate lines exhibited dramatically increased locomotor activity and maximal oxygen consumption, as well as increased mass-specific muscle cellular aerobic capacity, heart size, and hindlimb bone lengths (for review see Middleton et al. 2008). These effects were due to a Mendelian recessive allele that, when present in the homozygous condition, caused a 50 % reduction in hindlimb muscle mass (Garland et al. 2002). This gene has been mapped to a 2.6335-Mb interval on the MMU11 region of chromosome 11, which harbours ca. 100 genes that are likely to underlie muscle development and function (Hartmann et al. 2008). It must be noted, however, that despite threefold differences in voluntary wheel running, the selected and control lines do not differ with respect to BMR (Kane et al. 2008), which cautions against the existence of inescapable genetic link between BMR and aerobic capacity of skeletal muscles. This conclusion is corroborated by the lack of an effect of over-expression of mitochondrial uncoupling protein-1 (UCP-1) in skeletal muscles on BMR of HSA-mUCP-1 transgenic mice (Klaus et al. 2005). Interestingly, compared with littermate controls, HSA-mUCP-1 transgenic mice have substantially reduced levels of adiposity and increased total energy expenditures below the thermoneutral zone, most likely due to decreased muscle energy efficiency (Klaus et al. 2005). The lack of an appreciable effect of transgenic manipulation of UCP-1on BMR is puzzling and clearly deserves further study.

In contrast to small genetic differences underlying skeletal muscle function, Koch and Britton’s (2001) selection experiment resulted in a considerable between-line difference in gene expression of the heart muscle (Bye et al. 2008b). Out of 28,000 screened genes, 1,540 were differentially expressed between high (HCR)- and low (LCR)-running capacity lines. Interestingly, rats of HCR and LCR lines expressed genes underlying lipid and glucose metabolism, respectively. This suggests that the selection regime led to divergence in cardiac energy substrate utilization, towards mitochondrial fatty acid oxidation in HCR rats and glucose-based metabolism in LCR rats. Bye et al. (2008b) linked those differences in expression to genes coding uncoupling protein-4 (UCP-4) in the HCR line, which is likely to be involved in the regulation of fatty acid β-oxidation and therefore, influencing BMR.

The existence of an association between the effects of artificial selection on aerobic capacity and the genetics of heart muscle metabolism has also been confirmed by Babik et al. (2010) in a non-model rodent—the bank vole (*Myodes glareolus*). Babik et al. (2010) used 454 sequencing technology (for review see Wheat 2010) followed by expression profiling of the heart transcriptome in lines of bank voles selected for high metabolism as compared to unselected controls (Sadowska et al. 2008). They detected a number of putative single nucleotide polymorphisms (SNPs) between selection lines whose variant frequency differences were much higher than those expected by chance. Although the exact causal link between identified SNPs and the underlying response to selection on metabolic rate remains unclear, Babik et al.’s (2010) study exemplifies the potential offered by new generation sequencing technologies for studying BMR in animals whose genome sequences are not available (see also Vera et al. 2008).

## Conclusions and prospects

Our review shows that intra-specific variation in BMR remains a viable source of information regarding metabolic expenditure, with clear functional links to key metabolic processes at all levels of biological organization. We also expose a number of unanswered questions and emerging research areas, which we summarize below.

We have only briefly touched upon the discrepancies between the conclusions drawn from intra- and inter-specific studies on the significant factors affecting variation in BMR, such as the contribution of skeletal muscles (Raichlen et al. 2010) and fatty acid composition of the cell membranes (Polymeropoulos et al. 2012). Although the directionality of the correlations between BMR and those components do not need to be the same at the inter- and intra-specific levels, the lack of congruency is puzzling in the context of the proposed mechanisms of the evolution of endothermy (Nespolo et al. 2011). Most likely, this inconsistency can only be resolved by additional, within-species studies on animals from yet untapped parts of the inter-specific spectrum used in establishing patterns reported by Hulbert and Else (2005), Mitchell et al. (2007), Polymeropoulos et al. (2012) and Raichlen et al. (2010).

An accumulating body of information suggests that BMR is a heritable trait, at least under laboratory conditions. It would be therefore instructive to move quantitative genetics analyses of BMR into the field. With the advent of modern molecular genetic techniques, reconstruction of pedigrees for otherwise elusive species no longer poses an insurmountable difficulty, as evidenced by an increasing number of field studies on the traits such as fur coloration and flight metabolism (e.g. Nachman et al. 2003; Wheat et al. 2011). Such integration of molecular genetics with a conventional metabolic field studies would greatly strengthen the inference on adaptive significance of metabolic variation, so far based primarily on phenotypic data (for review see Whitehead 2012).

Likewise, the incorporation of functional genomics tools into studies on metabolic variation in the field is badly needed (Rokas and Abbot 2009). Borrowing from already well advanced biomedical research on gene expression, functional genomics should greatly advance the connections between metabolic phenotype, genotype and fitness in natural populations. As an initial blueprint, students of BMR functional genomics can follow already successful studies on morphological traits (e.g. fur coloration) as well as genomic studies on metabolic traits in insects (Wheat et al. 2011). Another, promising approach to identifying metabolic underpinnings of BMR is offered by metabolomics (Wone et al. 2011). Both targeted and untargeted global metabolic profiling of tissues and organs (Goodacre et al. 2004) can be used to generate and test the hypotheses regarding the physiological function underlying variation in BMR.

Our review shows that despite decades of research, the sources of intra-specific variation in BMR at organ, tissue and molecular levels are still not firmly identified. Paradoxically, in this regard, studies on free-ranging animals can be greatly illuminated by medical research that is quickly advancing thanks to the application of imaging technologies combined with genomics and other ‘omics’ research. While researching the literature for this review, we were struck by the poor exchange of information and ideas between researchers working on the physiology of metabolic rates within animals and humans. This also applies to studies on the major molecular pathways underlying BMR variation. We propose that ‘connecting the dots’ between metabolic studies on the whole-body level with mTOR activity holds promise for a grand picture integrating the regulation of the key metabolic mitochondrial oxidation, maintenance of membrane ionic gradients and biosynthetic costs, which together are manifested as energy expenditures quantified as BMR (Fig. 1).

## Abbreviations

AMPK: AMP-activated protein kinase
BAT: Brown adipose tissue
BMR: Basal metabolic rate (here corrected for the influence of body mass by ANCOVA)
DHA: Docosahexanoic fatty acid
H-BMR or L-BMR: Mice selected for high or low BMR, respectively
MMR: Maximum metabolic rate
MRI: Magnetic resonance imaging
mTOR: Mammalian target of rapamycin
PUFA: Polyunsaturated fatty acids
QTL: Quantitative trait loci
REML: Restricted maximum likelihood
SMR: Standard metabolic rate
SNP: Single nucleotide polymorphism
UCP: Uncoupling protein
